# A Screening Method Based on Headspace-Ion Mobility Spectrometry to Identify Adulterated Honey

**DOI:** 10.3390/s19071621

**Published:** 2019-04-04

**Authors:** María José Aliaño-González, Marta Ferreiro-González, Estrella Espada-Bellido, Miguel Palma, Gerardo F. Barbero

**Affiliations:** Department of Analytical Chemistry, Faculty of Sciences, University of Cadiz, Agrifood Campus of International Excellence (ceiA3), IVAGRO, P.O. Box 40, 11510 Puerto Real, Cadiz, Spain; mariajose.alianogonzalez@alum.uca.es (M.J.A.-G.); estrella.espada@uca.es (E.E.-B.); miguel.palma@uca.es (M.P.); gerardo.fernandez@uca.es (G.F.B.)

**Keywords:** ion mobility spectrometry, honey, adulteration, high fructose corn syrup, IMS Sum Spectrum, chemometrics, sensor

## Abstract

Nowadays, adulteration of honey is a frequent fraud that is sometimes motivated by the high price of this product in comparison with other sweeteners. Food adulteration is considered a deception to consumers that may have an important impact on people’s health. For this reason, it is important to develop fast, cheap, reliable and easy to use analytical methods for food control. In the present research, a novel method based on headspace-ion mobility spectrometry (HS-IMS) for the detection of adulterated honey by adding high fructose corn syrup (HFCS) has been developed. A Box–Behnken design combined with a response surface method have been used to optimize a procedure to detect adulterated honey. Intermediate precision and repeatability studies have been carried out and coefficients of variance of 4.90% and 4.27%, respectively, have been obtained. The developed method was then tested to detect adulterated honey. For that purpose, pure honey samples were adulterated with HFCS at different percentages (10–50%). Hierarchical cluster analysis (HCA) and principal component analysis (PCA) showed a tendency of the honey samples to be classified according to the level of adulteration. Nevertheless, a perfect classification was not achieved. On the contrary, a full classification (100%) of all the honey samples was performed by linear discriminant analysis (LDA). This is the first time the technique of HS-IMS has been applied for the determination of adulterated honey with HFCS in an automatic way.

## 1. Introduction

Motivated by the high demand for premium products and the elevated prices associated with them, food fraud is a common practice in the market [1]. The Food Standards Agency (FSA, UK), has defined this action as “deliberately placing food on the market, for financial gain, with the intention of deceiving the consumer” [2].

For this reason, it is of paramount importance to develop a rapid and reliable analytical method that allows us to identify adulterated food by routine control analysis, as it is the case for adulterated honey.

Honey is a natural food produced by bees. According to European Union Regulations (Codex Alimentarius Commission and Council Directive 2001/110/EC of 20 December 2001 relating to honey) honey is a pure product. Therefore, the addition or removal of any kind of substance is considered illegal [3]. Honey is a highly appreciated product for its superior nutritional properties and its numerous beneficial properties as an antioxidant [4,5] and anti-inflammatory [4,6]. Furthermore, it decreases cardiovascular risks [7] and presents prebiotic properties. Therefore, these well-known beneficial properties associated with honey are the reason why honey consumption has significantly increased in recent years [8]. In fact, imports from countries outside the European Union have been required due to the high demand. As a consequence of this, the price of high-quality honey has raised in the last few years [9]. Besides, some companies have started to adulterate honey by adding other products with a greater margin. Honey is most commonly adulterated by adding a cheaper sweetener (approximately 90%) [10]. High fructose corn syrup (HFCS) is one of the most commonly used adulterants because of its similar fructose content. Fructose is the main sugar found in honey, which makes it more difficult to detect it in adulterated honey [11].

Over the last years, Protected Designation of Origin (PDO) has been granted to honey to protect both, their geographical and their botanical origin [12]. PDOs are legal organizations of the European Union (EU) created in order to ensure compliance with EU Regulation No. 1151/2012. They guarantee that the products comply with the various geographical and quality criteria used by the protected designation.

Although a PDO should imply tighter control on honey quality, it also makes honey price rise according to other sweeteners [13]. Thus, honey adulteration is a fraud that still occurs in our society. This illegal activity is not only an economic fraud for companies and consumers, but also a hazard to people’s health because of the lack of control on allergenic products [14].

Different techniques have been used to identify adulterated honey [15,16] such as Nuclear Magnetic Resonance (NMR) spectroscopy [17], enzymatic activity [18], Raman spectroscopy [19], High-Performance Liquid Chromatography (HPLC) [20] or other DNA-based methods [21]. The methods based on those techniques have focused on determining specific compounds, which makes them easier to elude. In addition, their high cost and time requirements make them difficult to use as routine methods for food quality control. For this reason, in the last years, new methods based on the study of general profile signals avoiding identification of individual compounds have been developed. These types of methods are based on differences in the total signal from the volatile compounds, the total absorbance values of the compounds, etc. Thus, no individual quantification is needed. Methods in combination with multivariate analysis allow for a fast detection and discrimination of adulterated honey by performing characteristic fingerprints for each type of sample. General profile methods allow a fast and reliable characterization, do not require expert analysts, and allow for automatization. Some of the techniques applied for this purpose are the electronic tongue (e-tongue) [22] and electronic nose (e-nose) [23,24,25,26].

E-tongue and e-nose are analytical techniques for several compounds with a low limit of detection, low cost and based on fast methods. Due to their several advantages, both of them have been successfully applied for the detection of honey adulteration, for the characterization of botanical and geographical origin and even for different types of monofloral honey. However, these techniques present low selectivity and, therefore, they do not usually deal with the level of adulterants in the samples.

Ion Mobility Spectrometry (IMS) is an analytical technique for the analysis of volatile compounds (VOCs). Headspace above the sample is injected in the system, and then ionized at atmospheric pressure. The ionization can be carried out using an ultraviolet lamp, electrospray, a laser or by chemical ionization. Chemical ionization is one of the most commonly ionization used because of radioactive sources provide a stable and reliable operation. In addition, they do not require an external power source and have no maintenance requirements. ^3^H Tritium is one of the most applied isotopes for the chemical ionization [27].

The ionized analytes are separated inside a drift tube under the influence of a constant electric field. The time spent by each ion to cross the drift tube is known as drift time and it is characteristic of each analyte, since it varies depending on the size and geometric shape of analytes [28,29,30,31]. Once the ions reach the detector, the number of ions is translated in voltage units (intensity signal). 

Each drift time in the detector acts as a “sensor” and the total volatile compounds intensity collected at each drift time is equivalent to a multiple sensor signal. 

IMS can be used in two different ways on the determination of volatile compounds. On one hand, IMS can provide quantitative information about specific compounds. On the other hand, it can be also used as a fingerprint generator, it means, IMS can provide the spectra based on the volatile compounds allowing for the discrimination among different samples. IMS presents different advantages; it does not require solvents and the gas consumption is very low. For this reason, it is considered that methods developed based on this technique correspond to green analytical chemistry. Furthermore, IMS has a low limit of detection (ppb range), a fast response and a real-time monitoring. These features make of IMS a feasible technique for on-site analysis, since portable equipment can be used.

The possibility of real-time monitoring and the low limit of detection, accompanied by the feasibility of developing green methods, has increased the interest of analysts for this technique [27]. IMS may be coupled to other analytical techniques such as Headspace (HS) in order to concentrate VOCs, and/or to other separation techniques, such as Gas Chromatography (GC) or MS to operate in parallel. IMS has been successfully used in many different fields like drug or explosive detection [32,33,34] or for fire debris analysis [35]. It has also been used in several agro-food researches [36,37,38] and even for the detection of food fraud associated with oil [28,39], ham [28] or peanuts [40]. Despite the advantages of IMS and its successful application in food fraud and in other food-stuffs, to the best of the authors’ knowledge, there are no reports in the literature based on the use of IMS to identify adulterated honey.

Therefore, the aim of this study is to evaluate the feasibility of HS-IMS in combination with chemometric tools to detect HFCS added to honey samples at different percentages of adulterations.

## 2. Materials and Methods

### 2.1. Samples

The Regulation Council for Granada Protected Designation of Origin (PDO) (Lanjarón, Granada, Spain) provided the pure honey samples for this study. Thirty-three pure multi-floral honey samples (*n* = 33) harvested in 2016 were selected for the study. 

High fructose corn syrup (HFCS) was selected as the adulterant for this research, since it is one of the most commonly used products for this purpose. HFCS was purchased from a Spanish regular market (Cargill S.L.U., Martorell, Barcelona, Spain). 

The pure honey, the adulterant, and the adulterated samples were stored in absence of light at a room temperature of 25 °C in a store (4.05 m × 2.87 m × 2.28 m) prior to analysis.

### 2.2. Adulteration 

Multi-floral honey, since it is the most common type of honey, was selected as pure honey and to produce the adulterated samples [41]. To guarantee maximum heterogeneity, a 2 kg mixture made up of equal amounts from each of the 33 pure honey samples was prepared. This mixture was used as the unadulterated honey specimen and also to prepare the adulterated samples by adding the HFCS.

In general, adulterated honey in the market contains between 10% and 50% of adulterant, usually using HFCS, to be economically profitable but not easy to be detected. For this reason, mixtures of pure honey with added HFCS were prepared at the following ratios: 10%, 20%, 25%, 30%, 40%, and 50%. A total of 8 grams for each level of adulteration were prepared.

Samples of unadulterated honey (0%) and pure adulterant (100%) were also analysed. Two replicas of each level of adulteration were prepared. A total set of 16 samples: two pure honey samples, two adulterant samples, and 12 samples corresponding to the six adulterant-content levels. Furthermore, each analysis was carried out by duplicate.

In this way, samples were labelled as follows: firstly, adulterant content percentage; followed by the replica of the adulterations, i.e., 1 or 2; and finally, by the replica analysis for that sample, i.e., A or B. For instance, the first analysis of the first replica of adulterated honey sample with 10% adulterant content would be labelled as 10%_1_A and the second analysis of the second replica of adulterated honey samples with 25% adulterant content would be labelled as 25%_2_B.

### 2.3. HS-GC-IMS Analysis Acquisition 

The samples were analysed by Headspace-Gas Chromatography-Ion Mobility Spectrometry (HS-GC-IMS) Flavour Spec (G.A.S., Dortmund, Germany). The samples did not undergo any pre-treatments. The vials containing pure honey, HFCS or adulterated honey samples (0.1–0.9 g) were directly placed in the autosampler oven to be heated (30–50 °C) and agitated (500 rpm (5 s on and 2 s off)) in order to generate the headspace (HS). The GC column used was a multicapillary MCC OV-5 (5%-diphenyl, 95%-dimethylpolysiloxane) with dimensions of 20 cm × 0.2 µm) (G.A.S., Dortmund, Germany). The drift gas and the carrier gas selected were nitrogen with a purity of 99.999% obtained by means of a nitrogen generator (G.A.S., Dortmund, Germany). The ionization method used was ^3^H Tritium beta radiation.

The conditions related to HS were optimized by a Box–Behnken design. The conditions directly related to the GC-IMS analysis were as follows: EPC1 (Electronic Pressure Control of drift gas) was selected at its maximum level (250 mL/min) in order to avoid non-ionized compounds that might cause noises to interfere with the analysis. EPC2 (Electronic Pressure Control of carried gas) was selected as a ramp of 5 mL/min (t = 0 min), 10 mL/min (t = 5 min), and 25 mL/min (t = 10 min). The total analysis time was 15 min. The system temperature was set at T1: 45 °C; T2: 50 °C for HS conditions; T3 = T4: 80 °C.

HS-GC-IMS provided 2-dimensional data matrices (retention time vs. drift times). The IMS Sum Spectrum (IMSSS) was obtained by adding the total intensities of all volatile compounds at the different drift times regardless of the GC. Differences between the intensities of the IMS spectra from HFCS and pure honey could be used with the purpose of detecting any addition of HFCS to honeys. Nowadays, most of the devices where IMS technique is included are combined with GC. This combination ensures the ionization of all the analytes present in the sample and the correct identification of all of them avoiding competitive reactions which typically occur in IMS. 

**Figure 1:**
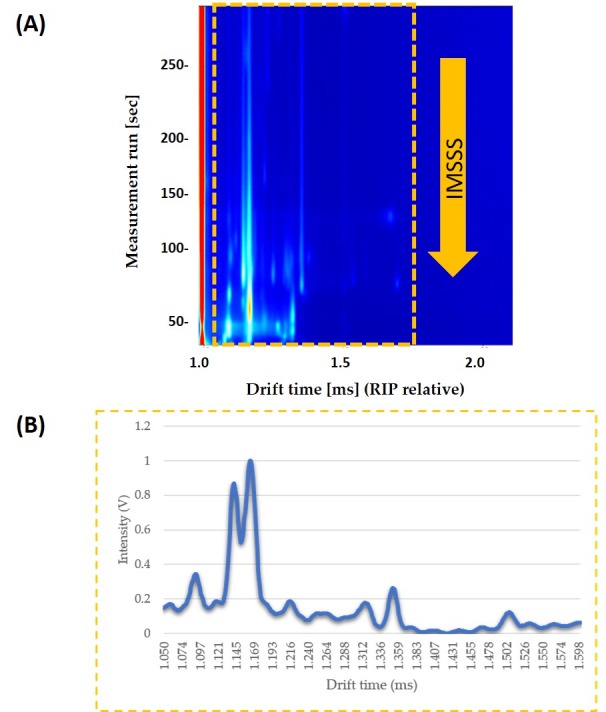
(**a**) Topographic plot of GC–IMS spectra obtained from pure honey sample under optimized conditions. The interested zone of each topographic plot is selected with a yellow rectangle; (**b**) The resulting IMSSS corresponding to the selected zone (intensity vs. drift time) displayed after summing the intensities across the chromatographic profile and so annulling the information about GC separation.

Each compound’s drift time was normalized to the signal of the Reaction Ion Peak (RIP). RIP was used as a reference signal, so that it is the signal of the water in the air ionized by tritium beta radiation and it represents the total of all the ions available for the ionization. IMSSS included the total intensity data from 4500 drift times, from 0.000 ms to 4.283 ms. Laboratory Analytical Viewer software (LAV) (G.A.S., Dortmund, Germany) was used to obtain the IMSSS for each sample. Once the IMSSS for each analysis was obtained, the area where all volatile compounds appeared was selected. The area was obtained by reducing the number of drift times to a total of 578 drift times (from 1.050 ms to 1.600 ms). Each range was normalized by assigning one unit to the maximum intensity (Figure 1).

The variables that affect the generation of the HS of the volatile compounds to be injected in the GC-IMS system were optimized. According to the literature, such influential variables are mainly incubation temperature, incubation time, injection volume and amount of sample [42]. Additionally, the pre-heating time for the honey to reach a regular temperature (30 °C) before the analysis was also studied. Thus, a total of five variables were selected for the optimization: incubation time, incubation temperature, injection volume, amount of sample and pre-heating time. For each variable, three levels were selected as shown in Table 1, coded as (−1) for the low level, (0) for the central point, and (1) for the high level. 

The ranges that have been evaluated for each variable were established according to the aim of this study, i.e., to develop a rapid analytical method to identify adulterated honey. For this reason, the incubation time and pre-heating time were limited to a maximum of 15 and 25 min, respectively.

The incubation temperature range was selected between 30 °C, since it was the minimum temperature allowed by the equipment, and 50 °C, since some honey components could suffer degradation at higher temperatures [43]. The injection volume was selected according to the instrument´s options. Small samples were selected based on the experience of the research group in the use of IMS with other food products.

Agitation was not selected as a variable to optimize. It was decided to fix it at 500 rpm in a period of 5 s of agitations and 2 s of stopping.

Box–Behnken design (BBD) in conjunction with response surface methodology (RSM) was applied to the optimization of the conditions to detect honey adulteration. The design consisted on 46 experiments with 6 repetitions in the central point (Table S1). All the trials were performed in random order.

In this case, the purpose was to maximize the differences between unadulterated honey and pure adulterant. Thus, only these two types of samples were used for the 46 experiences in the BBD. The total area of the IMSSS obtained for each sample under the specific conditions were analysed by the BBD (46 different conditions). The differences between the pure honey sample and the pure adulterant sample were calculated for any working condition. The optimization procedure tried to reach the conditions that showed the largest differences between the pure honey and the pure adulterant samples.

### 2.4. Data Analysis

For the analysis of the optimum conditions, Statgraphic Centurion XVI.I (Statgraphics Technologies Inc., The Plains, VA, USA) was used. 

Once the optimum conditions were determined, the samples that had been adulterated at different levels were analysed. Their IMSSS were combined with different chemometrics to study the suitability of this technique for the detection of adulterated honey. Hierarchical Cluster Analysis (HCA), Principal Component Analysis (PCA) and Linear Discriminant Analysis (LDA) were performed by using the statistical computer packages Statgraphic Centurion XVI.I and IBM SPSS Statistics 22 (Armonk, NY, USA). 

## 3. Results and Discussion

First of all, optimal conditions for the analysis of honey were determined. The use of IMS Sum Spectrum (IMSSS), which it is the sum of intensities of all volatile compounds across the chromatographic profile, was studied. This resulted in a spectrum with a total intensity for the different drift times. Once this method was optimized, IMSSS in combination with chemometrics, it was used to discriminate the different levels of honey adulteration by HFCS.

### 3.1. Optimization of the Method

One sample of unadulterated honey and one sample of pure HFCS under the different variable values were analysed. Their IMSSS were obtained and their spectra were reduced to the specific zone where the compounds associated with unadulterated honey and adulterant appeared. A spectrum from 1.050 ms to 1.600 ms with a total of 578 drift times was obtained. Each range was normalized by assigning one unit to the maximum intensity. The sum of the differences in intensities between the IMSSS from unadulterated honey and from HFCS in these 578 drift times was used as response variable for the BBD.

An Analysis of Variance (ANOVA) was carried out to evaluate the effects of the factors and the possible interactions between them. The results from the experiments with regards to intensity differences between pure honey and HFCS were correlated with those predicted by Equation (1).

Y = *β*_0_ + *β*_1_X_1_ + *β*_2_X_2_ + *β*_3_X_3_ + *β*_4_X_4_ + *β*_5_X_5_ + *β*_12_X_1_X_2_ + *β*_13_X_1_X_3_ + *β*_14_X_1_X_4_ + *β*_15_X_1_X_5_+ *β*_23_X_2_X_3_ + *β*_24_X_2_X_4_ + *β*_25_X_2_X_5_ + *β*_34_X_3_X_4_ + *β*_35_X_3_X_5_ + *β*_45_X_4_X_5_ + *β*_11_X_1_^2^ + *β*_22_X_2_^2^ + *β*_33_X_3_^2^ + *β*_44_X_4_^2^ + *β*_55_X_5_^2^ (1)

Equation (1) corresponds to a quadratic model where Y is the predicted response (predicted difference between unadulterated honey and adulterant at the different drift times); *β*_0_ is the model constant; X_1_ (time for headspace generation), X_2_ (temperature of the headspace), X_3_ (injection volume), X_4_ (amount of sample), and X_5_ (pre-heating time) are the independent variables; *β*_i_ are the linear coefficients; *β*_ij_ are the cross-product coefficients, and *β*_ii_ are the quadratic coefficients.

The results from the analysis are shown in Table 2. The coefficients for the different parameters of the quadratic polynomial equation and their significance (*p*-values) are presented. The factors that showed a *p*-value lower than 0.05 were considered to be significant factors that influenced the response at the selected level of significance (95%). 

As can be observed, incubation time, incubation temperature, injection volume, amount of sample and quadratic interaction of the injection volume are influential variables (*p*-values < 0.05). Incubation temperature and injection volume showed positive coefficients (*b_2_* = 0.0370 and *b_3_* = 4.5332) which means that the discrimination between the two groups was more efficient when the incubation temperature and the volume of the injection were increased.

**Figure 2:**
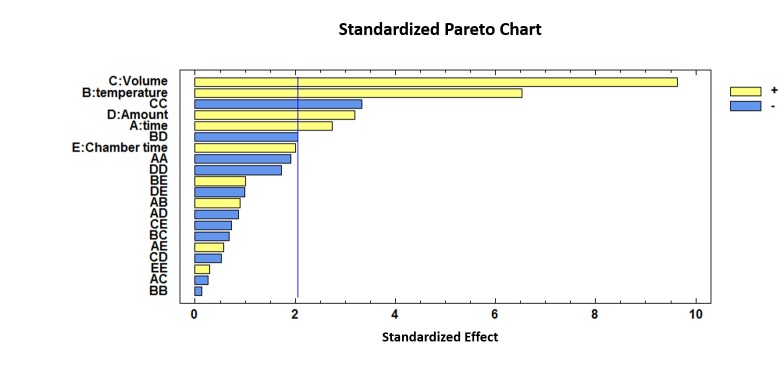
Standardized Pareto Chart representing the five variables optimized for the discrimination between unadulterated honey and HFCS (high fructose corn syrup) samples.

For a visual representation of the effects and their combinations, a standardized Pareto Chart is depicted in Figure 2.

The correlation was evaluated by means of the squared correlation coefficient (R^2^). In this case a value of R^2^ = 87.95% was obtained, which indicates a statistically significant agreement between the measured and the estimated responses. 

**Figure 3:**
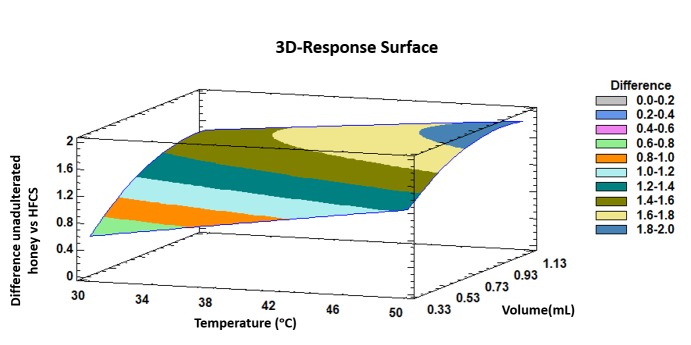
3D surface plot of the Box–Behnken design that represents graphically, according to the polygonal equation, the influence of temperature and injection volume on the intensity differences between unadulterated honey samples and HFCS samples.

The trends outlined above were recorded in a three-dimensional surface plot obtained by applying the polynomial equation. Figure 3 illustrates the combined effects of temperature and injected volume on the differences between unadulterated honey samples and HFCS samples.

It can be observed that both, incubation temperature and injection volume at the highest levels of the evaluated ranges show the maximum intensity differences between the two groups of samples.

Based on previous bibliography and the experience of the research group, it was expected that the maximum discrimination was achieved with the maximum injection volume. However, in the case of IMS competitive ionization reactions can occur, so an increment of the volume does not always ensure larger differences among the samples. In addition, too much sample volume can break the RIP in the IMS. Additionally, it is also important to study the linearity of the influence of the volume. Therefore, it was important to optimize this variable. As it can be observed in low terms the effect of the injection volume on the discrimination is linear but at high levels, the effect starts to be curved. 

The final optimum conditions to discriminate between unadulterated honey samples and HFCS samples were as follows: 0.15 g of sample analysed, 15 min incubation at 50 °C with 22 min of pre-heating time and an injection volume of 0.91 mL.

### 3.2. Repeatability and Intermediate Precision of the Method

The analytical properties of the new method were established, specifically, repeatability and intermediate precision. Repeatability was calculated as closeness between the results of the experiments completed on the same day under the same conditions. Intermediate precision was calculated as the closeness between results on different days. A total of 12 analyses were carried out under optimum conditions, for each of the two samples (unadulterated honey and HFCS). Repeatability was studied by performing six analyses on the same day. Intermediate precision was evaluated by performing other three analyses during the following two days. A total of 24 samples were analysed (12 samples of unadulterated honey and 12 samples of HFCS).

The sum of the intensity differences between samples of unadulterated honey and HFCS in repeatability and intermediate precision experiences was calculated. The Coefficient of Variation (CV) between the sum of the intensity differences was used as the variable to measure their similarity.

The repeatability was 4.90%, and the intermediate precision 4.27%. Since both values were within the acceptable limits (5%), the developed method proved to be repeatable and with an adequate intermediate precision.

### 3.3. Analysis of the Adulterated Honey Samples

Once the method had been optimized, it was applied to determine the level of HFCS on a set of adulterated honey samples. Two replicas of each of the 16 samples (two unadulterated honey, 2 HFCS and two samples at each percentage of adulteration; i.e., 10%, 20%, 25%, 30%, 40%, and 50%) were analysed by HS-IMS under the previously determined optimum conditions. 

Normalized spectra from 1.050 to 1.600 ms were obtained for each sample in order to study the spectra profile of each group. Differences in total intensities for each drift time were detected at the different percentages of adulteration (information not included). The next step was the evaluation of the possibility of using these differences to discriminate between adulterated honey levels and unadulterated honey without identifying individual compounds. 

**Figure 4:**
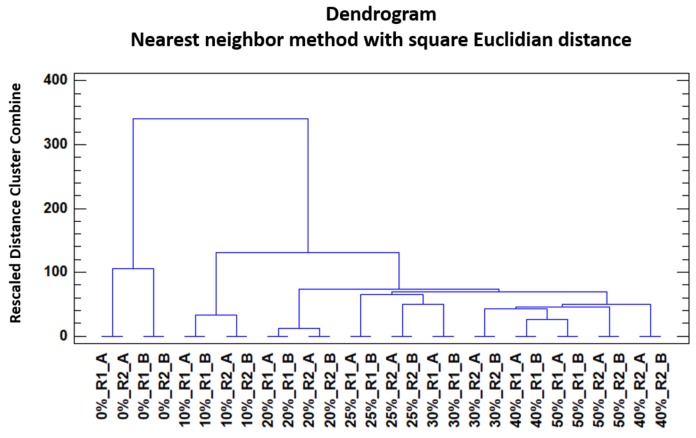
Dendrogram obtained by means of HCA of the unadulterated honey and the adulterated honey samples (D_28X578_).

In order to study the trends of the honey samples to get grouped according to their level of adulteration by HFCS, a non-supervised method was selected. HCA and a Nearest neighbour method with square Euclidian distance was applied. For this analysis, the IMSSS of the two replicas from each of the 14 samples (two unadulterated honey and two samples of adulterated honey at each one of the different percentages) were analysed by HCA. The results were graphically represented in a dendrogram (Figure 4).

It can be seen that samples tended to group depending on their adulterant content and percentage. Unadulterated honey samples formed a separate cluster at a considerable distance from the rest of the adulterated samples, which also formed different groups. It could be seen that the adulterated samples also had a tendency to form groups depending on their percentage of adulterant. Nevertheless, and despite this tendency, there were also some misclassifications with regards to the percentage of adulterant content. These misclassifications were more noticeable in samples with an adulterant content over 25% 

**Figure 5:**
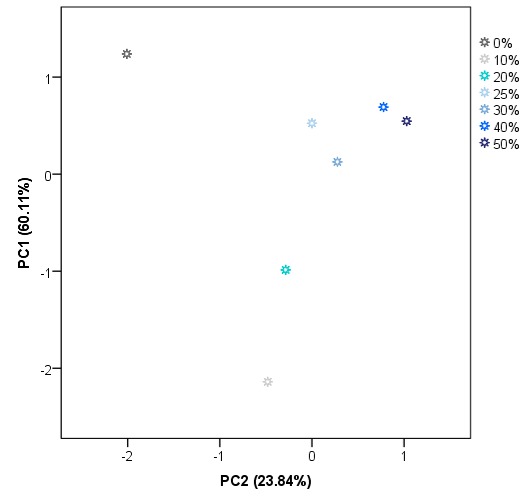
Two-dimensional PCA centroids plot (scores average for PC1 vs PC2).

These results suggest that IMSSS differences are related to some of the compounds that can be found in adulterated honey and also to their percentage in the sample. However, a successful and complete classification of the samples was not achieved. Therefore, another non-supervised technique namely PCA was applied to the same data matrix D_28X578_. Principal components (PCs) with eigenvalues greater than 1 were extracted, obtaining a total of 8 PCs. PC1 accounts for 60.11% of the variance of the IMSSS data. The first two factors account for more than 83.95% of the total variance and the first three more than 96%. The score plot for group centroids according to the first two PCs is shown in Figure 5.

As can be observed, PC1 seems to be most related to the level of adulteration. A percentage of 10% and 20% of adulteration centroids presented negative values for PC1. In general, centroids for the highest level of adulteration groups also present the highest values for PC1. On the other hand, PC2 is mainly related to the presence/absence of adulteration but it is also necessary for the classification regarding the level of adulteration. As Figure 5 illustrates, pure honey centroid presented the highest negative value for PC2. Centroids of low levels (10%, 20%, and 30%) also present negative values for PC2, whilst centroids of adulterated samples at higher levels have positive values on this PC. These results suggest that there is a tendency of the centroids to be classified on the space according to the level of adulteration based on the PC1 and PC2. As in the case of HCA, a perfect classification based on the presence of adulteration but not on the level of adulteration was achieved. The misclassification is again in the case of centroids above 25% of adulteration). As a full classification of the samples was not achieved by using unsupervised techniques, it was proceeded with a supervised technique, in particular, LDA. 

**Figure 6:**
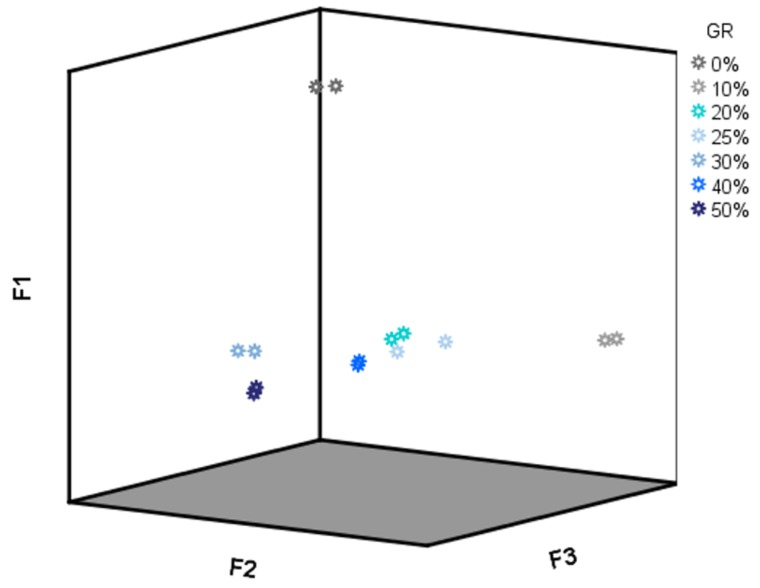
F1, F2 vs F3 score plot (D28X578).

As PC1 and PC2 showed high loading values (higher than 0.8) for almost all of the drift times (data not shown), a specific region for the LDA was not selected for this analysis but the whole data matrix D_28X578_. All of the 28 IMSSS were subjected to LDA using a cross-validation and stepwise method. Six groups were established a priori according to their adulterant content percentage (0–50%); 100% discrimination was obtained for all the groups. Discriminant scores for all samples according to the first three discriminant functions (F1, F2 and F3) were represented in Figure 6. As it could be observed, F1 is the main responsible for the discrimination between unadulterated and adulterated honey samples. However, F2 and F3 are necessary for the discrimination between the different percentages of adulteration. Based on the LDA results, adulterated honey samples can be identified, and their percentage of adulteration can be discriminated based on their IMSSSs.

## 5. Conclusions

This study has optimized, for the first time, a method based on analytical ion mobility spectrometry coupled to headspace for the identification of adulterated honey. Five relevant variables: incubation temperature, incubation time, injection volume, amount of sample and pre-heating time, have been optimized. IMSSS, as a novel data treatment, has been proposed to optimize the analysis of adulterated samples. IMSSS would allow the comparison of data between laboratories in a rapid and reliable way. 

Incubation time, incubation temperature, injection volume, amount of sample and the quadratic interaction of the volume of the injection with itself were the most influential variables. The optimum conditions were determined as follows: 0.15 g of sample, 22 min pre-heating time, analysed at 50 °C, for 15 min and with 0.91 mL injection volume. The method evidenced high repeatability and intermediate precision with RSDs below 5% in both cases.

The optimum conditions for the developed method have been applied to adulterated samples of multi-floral honey adulterated with HFCS at different percentages (10%, 20%, 25%, 30%, 40%, and 50%). 

IMSSS combined with chemometrics, such as HCA, PCA and LDA has demonstrated to be a suitable tool for the identification of multi-floral honey adulterated by adding HFCS and even to discriminate different adulteration levels depending on the adulterant content. A full classification was achieved for all samples in the LDA.

Based on these satisfactory results, further research studies should study the applicability of this method to other honey adulterants and to other honey varieties. 

## Figures and Tables

**Table 1:** Selected variables, their values and coded and un-coded levels used for the BBD.

Variable	−1	0	1
Incubation time (min)	5	10	15
Incubation temperature (°C)	30	40	50
Injection volume (mL)	0.33	0.66	1.00
Amount of sample (g)	0.1	0.5	0.9
Pre-heating time (min)	5	15	25

**Table 2:** Analysis of variance of the quadratic model adjusted to the discrimination of pure honey and HFCS samples.

Variable	Factor	Coefficient	Sum of Squares	Degrees of Freedom	Mean Square	*F*-Value	*p*-Value
Time	X_1_	0.0601	0.1975	1	0.1975	7.51	0.0111
Temperature	X_2_	0.0370	1.1216	1	1.1216	42.67	0.0000
Injection volume	X_3_	4.5332	2.4363	1	2.4363	92.69	0.0000
Amount of sample	X_4_	3.4407	0.2684	1	0.2684	10.21	0.0038
Pre-heating time	X_5_	−0.0167	0.1057	1	0.1057	4.02	0.0558
Time: Time	X_1_^2^	−0.0042	0.0955	1	0.0955	3.63	0.0682
Time: Temperature	X_1_X_2_	0.0014	0.0209	1	0.0209	0.80	0.3800
Time: Injection volume	X_1_X_3_	−0.0128	0.0018	1	0.0018	0.07	0.7931
Time: Amount of sample	X_1_X_4_	−0.0348	0.0194	1	0.0194	0.74	0.3985
Time: Pre-heating time	X_1_X_5_	0.0009	0.0085	1	0.0085	0.32	0.5746
Temperature: Temperature	X_2_^2^	−0.0001	0.0004	1	0.0004	0.02	0.9002
Temperature: Injection volume	X_2_X_3_	−0.0163	0.0118	1	0.0118	0.45	0.5076
Temperature: Amount of sample	X_2_X_4_	−0.0416	0.1109	1	0.1109	4.22	0.0505
Temperature: Pre-heating time	X_2_X_5_	0.0008	0.0264	1	0.0264	1.00	0.3263
Injection volume: Injection volume	X_3_^2^	−1.6303	0.2919	1	0.2919	11.11	0.0027
Injection volume: Amount of sample	X_3_X_4_	−0.3161	0.0072	1	0.0072	0.27	0.6059
Injection volume: Pre-heating time	X_3_X_5_	−0.0175	0.0138	1	0.0138	0.53	0.4752
Amount of sample: Amount of sample	X_4_^2^	−0.5930	0.0786	1	0.0786	2.99	0.0962
Amount of sample: Pre-heating time	X_4_X_5_	−0.0200	0.0256	1	0.0256	0.98	0.3329
Pre-heating time: Pre-heating time	X_5_^2^	0.0002	0.0022	1	0.0022	0.09	0.7724
Pure error		−2.9622	0.6571	25			
Total		0.0601	5.4518	45			

## Supplementary Materials

The following are available online at https://www.mdpi.com/1424-8220/19/7/1621/s1, Table S1: Box–Behnken design experiments.
