# Supplementary material for: A Screening Method Based on Headspace-Ion Mobility Spectrometry to Identify Adulterated Honey
Source: Sensors (Basel). 2019 Apr 4;19(7):1621. doi: 10.3390/s19071621 (PMC6480427; doi:10.3390/s19071621)
Supplement: Supplementary file 1 [file sensors-19-01621-s001.pdf]

A Screening Method Based on Headspace- Ion Mobility Spectrometry to Identify Adulterated Honey

María José Aliaño-González, Marta Ferreiro-González*, Estrella Espada-Bellido, Miguel Palma and Gerardo F. Barbero.

Department of Analytical Chemistry, Faculty of Sciences, University of Cadiz, Agrifood Campus of International Excellence (ceiA3), IVAGRO, P.O. Box 40, 11510 Puerto Real, Cadiz, Spain

* Correspondence: marta.ferreiro@uca.es; +34 956 016359.

Table 1. Box-Behnken design experiments.

Exp.	Variables					Difference
	**Incub. Time (min)**	**Incub. Temperature (°C)**	**Volume of Injection (mL)**	**Amount of Sample (gr)**	**Pre-Heating Time (min)**	
1	−1	−1	0	0	0	1.301
2	0	1	0	1	0	1.627
3	0	−1	0	−1	0	0.605
4	0	0	0	0	0	1.482
5	0	−1	1	0	0	1.458
6	1	0	−1	0	0	0.672
7	0	1	0	0	−1	1.635
8	0	−1	0	0	1	1.191
9	0	0	1	−1	0	1.509
10	−1	0	0	0	−1	1.173
11	0	0	−1	1	0	1.096
12	0	0	0	1	−1	1.442
13	1	0	0	−1	0	1.555
14	0	1	−1	0	0	1.298
15	0	0	0	0	0	1.500
16	0	0	1	0	−1	1.787
17	0	0	0	1	1	1.558
18	−1	0	0	1	0	1.453
19	0	0	0	−1	1	1.340
20	0	0	0	0	0	1.482
21	−1	1	0	0	0	1.497
22	0	0	1	1	0	1.727
23	1	0	0	1	0	1.513
24	1	0	0	0	1	1.651
25	0	−1	0	1	0	1.199
26	−1	0	0	0	1	1.190
27	0	1	1	0	0	1.881
28	−1	0	1	0	0	1.386
29	0	1	0	0	1	1.844
30	0	0	−1	−1	0	0.707
31	0	0	−1	0	1	1.230
32	1	−1	0	0	0	1.259
33	0	0	−1	0	−1	0.889
34	−1	0	−1	0	0	0.410
35	−1	0	0	−1	0	1.216
36	1	0	1	0	0	1.563
37	0	−1	0	0	−1	1.307
38	0	0	0	0	0	1.482
39	1	0	0	0	−1	1.449
40	0	1	0	−1	0	1.699
41	0	0	0	0	0	1.482
42	0	0	0	0	0	1.482
43	0	0	0	−1	−1	0.904
44	0	−1	−1	0	0	0.658
45	0	0	1	0	1	1.892
46	1	1	0	0	0	1.745
